# Mobility, Out-of-Home Activity Participation and Needs Fulfilment in Later Life

**DOI:** 10.3390/ijerph16245109

**Published:** 2019-12-14

**Authors:** Susanne T. Dale Nordbakke

**Affiliations:** Institute of Transport Economics, Gaustadalléen 21, NO-0349 Oslo, Norway; Susanne.Nordbakke@toi.no; Tel.: +47-48-95-94-18; Fax: +47-22-60-92-00

**Keywords:** aging, mobility, out-of-home activity participation, preferences, needs fulfilment, wellbeing

## Abstract

Using nationally representative survey of 4723 people aged 67 or older living in Norway, this paper explores the link between wellbeing, out-of-home activity participation and mobility in old age. A basic assumption of this paper is that out-home activities mediated through mobility can contribute to needs fulfillment, and, hence, wellbeing. This study explores the role of preferences, and individual and contextual constraints, in both the overall level of out-of-home activity participation in old age and the level of participation in three specific out-of-home activities (grocery shopping, visiting family or friends, and attending cultural activities). A person’s degree of home orientation is used as an indicator of preference for indoor activities. The findings suggest that age, living status, income, education, holding a driving license, health, social network, centrality of residence, and the quality of the public transport supply have a significant impact on the overall participation level. In addition, the study suggests that the types of constraints vary between travel purposes and the location of activities. Moreover, there is an independent effect of the degree of home orientation on the overall participation level, on the degree of visits to family and friends and on the degree of attending cultural activities, which suggest that people differ in their need for out-of-home activities. However, degree of home orientation has no impact on the degree of grocery shopping, which might imply that grocery shopping is more independent of preferences. The main conclusion from this study is that the extent to which out-of-home activities fulfill needs vary between individuals, depending on their preferences as well as the interplay between individual abilities and resources and contextual conditions.

## 1. Introduction

Older people in developed countries are healthier, wealthier and live longer than ever before, and the population in these countries is ageing. Older people today also travel more than previous generations [1,2,3,4]. Nevertheless, as people age, they are more likely to experience problems with transport and daily travels (see, e.g., [5,6,7]). Although many conditions strike people much later than they did a few decades ago, increased longevity implies that problems with transport will concern a large number of people in the next decades, especially the oldest old (age 80 and older).

Being able to move around and about is important for independent living in old age, and for the ability to meet and interact with relatives and friends to pursue other meaningful activities [8]. Several studies have shown that travel in daily life, and being able to drive and to participate in out-of-home activities, are positively linked to wellbeing in old age [9,10,11,12,13], even though the conceptualization and operationalization of wellbeing varies greatly among these studies [14]. 

For a society to provide for independent travel in old age, it needs to understand the travel needs and desires of older people and the options for and constraints on mobility and out-of-home activities that they encounter when ageing. 

In transport research, travel is primarily seen as a derived demand [15,16], based on an individual’s needs and desires to participate in activities away from home. A basic assumption in this paper is that out-of-home activities mediated through mobility can contribute to needs fulfilment, and hence wellbeing, as understood by the Finnish sociologist Erik Allardt [17]. The paper explores potential explanations for out-of-home activity participation in general and in three different out-of-home activities—grocery shopping, visiting family and friends and attending cultural activities. Out-of-home activity participation is used as an indicator of needs fulfilment.

The paper will contribute to the literature in several ways. While most of the research on mobility and out-of-home activity participation in old age has explored potential individual or contextual constraints, this paper will also explore the role of preferences in explaining differences in out-of-home activity participation and needs fulfilment in old age. Although prior research has shown that actual out-of-home activity participation is positively linked to lower subjectively reported unmet needs for out-of-home activities [17], there might be variations among people in the extent to which they can participate in out-of-home activities. Moreover, most prior research has studied general mobility, trip frequency and out-of-home activity participation in old age; this paper will explore whether potential explanations for participation differ between types of out-of-home activities, that pertain to different types of needs. Empirical research on whether or not factors associated with out-of-home activity participation in old age vary with travel purposes and destinations is limited.

### 1.1. Wellbeing, Needs Fulfilment and Out-of-Home Activities

According to Allardt [17], wellbeing amounts to the fulfilment of needs along three dimensions:

- To have: the material level of living and the need for material resources (for example, work, education and money);
- To love: the non-material aspects of life—more specifically, the need for social relations, such as friendship and family ties;
- To be: the need for self-realization and positive judgment of oneself (which might be fulfilled through such things as education, work or friendships)

The satisfaction of needs along one dimension can often, in itself, be a resource that could be mobilised to satisfy other needs along the same dimension or along other dimensions. Hence, the satisfaction of needs along these dimensions can be understood both as outcome (result) and input (resource). For example, education can both satisfy the need to be (as it contributes to self-realization) and function as a resource in obtaining employment, which, in turn, can fulfil the need *to have* (e.g., income) and, in many cases, also the need *to be* (e.g., self-realization through work). For Allardt [17], needs are socially defined and can change; there is no generic list of needs for all times and places. However, he also argues that, at least in certain societies and groups, there exists a modicum of agreement on the most important needs.

The basic assumption in this paper is that out-of-home activities can contribute to the fulfilment of needs along the dimensions to have, to love and to be, and that mobility can contribute to the fulfilment of these needs. Put simply, shopping trips, service trips (including those for medical purposes) and commuting are indicators of *having*; visiting trips and chauffeuring are expressions of social interaction—of *loving*; and trips to access different types of leisure activities can be seen as indicators of *being.* In many cases, participation in one specific activity can fulfil needs along several dimensions, e.g., going to the cinema together with friends can fulfil needs associated with loving and being. It is evident that participating in out-of-home activities is not the only way in which Allardt’s needs can be fulfilled; in-home activities are also very important. Moreover, the extent to which needs can be fulfilled through activity participation probably varies between individuals, depending on their preferences, desires and personality. In addition, people may also adjust their ambitions and expectations regarding what they need to the capacities and resources they can mobilise. Hence, it is important to depart from an individual point of view when exploring the degree to which needs are fulfilled in terms of out-of-home activity participation. 

In the next section, a theoretical framework for understanding mobility and out-of-home activity participation in old age is introduced and a short overview on the existing knowledge on the factors associated with mobility, out-of-home activity participation and needs fulfilment in old age is presented. 

### 1.2. Understanding Mobility and Out-of-Home Activities in Old Age

#### 1.2.1. Mobility and Out-of-Home Activity Participation: A Result of Preferences and Opportunities

A common approach to understanding individual travel is that travel between two geographically distributed places is often understood as a result of an individual’s needs and desires, modified by a series of individual and contextual constraints, most often understood as spatial constraints [18,19,20]. In order to make the importance of choice more explicit in regard to travel and out-of-home activity participation in old age, and to explore the role of contextual constraints beyond spatial constraints, this paper will apply a classical sociological approach to action, as understood by the philosopher and social scientist Jon Elster [21]. He perceives action as the result of the choices made—according to an individual’s preferences—from given opportunities for action or, as he puts it, “by what they can do and what they want to do” [21]. In this paper, opportunities for mobility—or action—are understood as the interplay between an individual’s resources and ability for mobility, modified by the constraints and opportunities given in the contextual conditions for mobility. An individual’s ability for mobility, as defined in this paper, can comprise all those capacities and resources an individual holds that promote mobility in old age, such as physical, material, temporal and social resources, in addition to competence and skills in regard to mobility and out-of-home activity participation. Physical resources refer to an individual’s functional condition, such as their general health condition and walking ability. Material resources refer to finances and access to individual transport resources (access to a car, holding a driving license, having a bike), which are often interlinked, while the available time for transport and participation in activities is a temporal resource [22]. Social resources refer to having a social network of family and friends that can help with transport and/or that can accompany a person in activities. When studying individual ability for mobility in old age, background factors such as age and gender are also important, as these often are highly correlated with individual ability for mobility. 

Contextual conditions for mobility are defined in this paper as the social, temporal and spatial attributes of the context (of the trip itself or the desired out-of-home activities), which either promote or hinder an individual’s mobility. Spatial conditions have received most attention within urban planning and geography [20]. The spatial conditions refer to the land use component (reflecting the availability and quality of facilities and activities) and transportation (reflecting the availability and quality of the transport system). These components are often related to the residential location of an individual. The temporal conditions refer to the temporal organization of activities and the transport supply (e.g., shopping hours, time schedules for public transport supply in the weekends, etc). Social conditions encompass macro-level social factors, such as level of affluence in society or prevailing values and regulations (e.g., age limitation for driving), but also micro-level factors, such as the social environment of a trip made (e.g., other passengers on a bus, crime rate at the residential location) or at the desired destination of a trip.

Opportunities for mobility and actual mobility are not necessarily directly related. Some people do not travel, even when they have access to a great variety of activities and good access to individual resources and abilities for mobility (e.g., time, health, transport resources) because they do not have a need or desire to participate in these activities. Others might travel only to secure their most basic needs (e.g., food, health checkups). On the other hand, poor accessibility (located far away or not accessible by public transport) does not necessarily imply non-participation in an activity. The strength of the desire to participate in an activity might play a crucial role. People are different in terms of preferences and desires for out-of-home activity participation, and in terms of what kind of activities outside the home they would like to participate in. 

However, preferences for out-of-home activity participation can change with age. They change and shift over time, often in relation to the individual’s context and the perceived level of resources that can be mobilized in order to enact certain behaviours (including trip making and activity participation). People may have become strongly oriented towards their home in response to (more) limited options or stringent constraints.

#### 1.2.2. Preferences for Mobility and Out-of-Home Activity Participation

Research on the role of preferences for mobility in old age seems scarce; only one study has been identified, but this has not studied the role of choices explicitly: in a multivariate analysis based on data from five European countries, Mollenkopf et al. [23,24] explored, amongst others, the effect of outdoor–indoor motivation, measured by the extent to which respondents’ perceived themselves as an outdoor- or indoor type, on the variety of outdoor activities pursued by people age 55 or older. They found that outdoor–indoor motivation, along with an individuals’ health, income, education, social network and high-quality neighbourhood features had a significant impact on the variety of outdoor activities pursued. However, they did not control for holding a driving license. 

The main objective of the present study is to explore whether older people choose differently in terms of the degree of mobility—according to their preferences—within a given set of opportunities (both individual resources and abilities and contextual conditions for mobility).

In the following sections, previous research on factors associated with mobility, more detailed objectives and a hypothesis of this study will be outlined. 

#### 1.2.3. Individual Resources and Abilities 

The number of trips made for out-of-home activities decreases with age [1,2,25]. Work-related trips cease after retirement, and mobility is likely to be reduced with age as the number of impairments increases. In a German study of people aged 60 or over [26], ability to move was found to be one of the most important predictors for out-of-home leisure activities.

It is often suggested that not having a driving license has an impact on older women’s level of out-of-home activity [27,28]. Although the gap between the proportion of older men and women who hold driving licenses is decreasing, the share of older women holding a driving license is still lower [1,3,5] and women tend to give up driving earlier than men [28,29]. However, research has shown that the probability of continuing to drive (as opposed to giving up driving) in old age is strongly associated with a long and active driving career, both among older people in general [30] and among older women [31]. 

The results from studies of the role of gender in mobility in old age are contradictory. Both Marottoli et al. [11] and Scheiner [26] found that men reported significantly lower activity levels than women; Schwanen et al. [27] found that women are less active than men in leisure activities when controlling for various background factors; Siren and Hakamies-Blomqvist [28] found that older women have lower levels of out-of-home activity participation than men. However, when controlling for gender, age, educational level and place of residence, Siren and Hakamies-Blomqvist found that only holding a driving license and residential location had a significant effect on out-of-home activity participation.

It can be argued that the significance of a driving license for mobility might be related to a spurious effect of better health among car holders. Empirical findings show that respondents with a driving license are likely to be healthier than the rest of the older population [32,33]. Still, in a longitudinal study of men and women age 65 years and older, Marottoli et al. [11] showed that driving cessation was strongly associated with reduced out-of-home activity levels, even after adjusting for health and socio-demographic factors. This is unlike Scheiner’s study [26], from Germany, that found no influence of car availability on the household or on trip frequency for leisure activities, nor for unmet leisure activity wishes, when controlling for age, health, gender, and place of residence. However, two more recent studies from the European context, respectively Denmark and Norway, have demonstrated the significant impact of holding a driving license on the extent to which older people believe that their needs for out-of-home activities remain unfulfilled, even when controlling for health, socio-demographics and residential location/population density [14,34]. The difference in the findings in the German study compared to the Danish and Norwegian study might relate to contextual differences (e.g., settlement structures and quality of the public transport supply in rural areas). Nevertheless, previous studies have shown that older people do value having access to a car and holding a driving license. A study from Sydney, Australia, showed that the preference to continue driving remains strong as individuals age, even if public transport and/or being a car passenger are available options [35], and a study in London showed that older people prefer driving to public transport [36]. This might be explained by the compensatory qualities that the car/holding a driving license entails, as suggested by some researchers [8,28]. This claim is supported by an analysis of the Norwegian travel surveys [6], which found that common age-related health conditions have greater effect on walking and using public transport than on the use of a car. 

But being able to hold a car in the household might also relate to financial resources. Moreover, it is likely that the ability to travel is greater if one can afford taxis. Research has shown that income per person has a significant impact on trip frequency among people aged 55 and above in five different countries [24]. Education is often highly associated with income, and Schwanen et al. [27] found, in their study of people aged 50 and above, an effect of education and being gainfully employed on trip frequency, when controlling for other factors. 

Some studies have found a significant effect of a social network of family and friends on trip frequency and out-of-home activity participation, when all else is equal, among older people [26,27]. These findings might suggest that, with a social network, it is easier to get help for transport to activities. They might also suggest that having a large network of family and friends increases the possibility of having company for activities. Although company for activities is not a precondition, as some activities can be enjoyed without company (e.g., going to the theater) it is likely that some activities are more enjoyable with company. Living in a partnership might also be indicative of both a transport resource (help with transport when having a partner who drives a car) and access to company. However, both Schwanen et al. [27] and Scheiner [26] have found that living alone has positive effect on trip frequency/out-of-home activity participation in old age, when all else is equal. This has been explained by a greater need for social activities outside the home when living alone compared to living with a spouse/partner. 

In this study, the impact of health-related factors, income, holding a driving license, living status and a social network on the level of out-of-home activities is explored. One specific objective is to explore the role of the car/holding a driving license for the level of out-of-home activity participation in old age in a Norwegian context. 

Another objective of the study, is to explore various constraints for different travel purposes – grocery shopping, visits to family and friends and cultural activities, which is assumed to fulfill different needs (respectively “having”, “loving”, and “being”). It is also assumed that the constraints for participation in these three different activities will vary. It is hypothesized that the need for a car differs for specific travel purposes, depending on the geographic location of the activities selected for analysis: grocery stores and supermarkets are often located in the neighbourhood, within walking distance. Family and friends may live in the neighbourhood and/or in another part of the municipality. Cultural activities, such as cinema, theatres and restaurants, are likely to be located in the central areas of the municipality/town/city. Public transport tends to be best suited for trips into the centre of a city/town; it is less well suited to crisscross travel (e.g., from suburb to suburb) and least suited for trips across rural areas. 

#### 1.2.4. Contextual Conditions for Mobility

Several studies have shown that living in high-density areas has a positive effect on trip frequency in old age [27,28] when controlling for other socio-demographic factors; this might be explained by proximity to destinations and the better public transport supply in urban areas. In this study, the effects of residential location and the quality of the transport supply in the residential area of an individual are also explored. 

In this paper, the general degree of out-of-home activity participation is used as an objective indicator of the general level of needs fulfilment, and, hence, wellbeing. The major contribution of the study to the literature, is to answer the question of whether people with the same types of constraints have different preferences, and whether constraints and preferences differ between different types of activities. 

## 2. Material and Methods

### 2.1. Participants

A questionnaire was sent out to 12,000 people age 67 (the retirement age in Norway). The sample was randomly drawn from a national representative population database owned by KANTAR, a Norwegian survey company. The database includes only people living in their private homes and not those living in institutions. The survey covers all parts of the country and cities, suburbs and rural areas. 

The survey was conducted from 22 October to 23 November 2011. A reminder, with a copy of the questionnaire, was sent after one week. The response rate was 40 percent (N = 4723). More than 300 of the non-respondents were reported (by telephone or letter) to be unable to answer because of dementia, a move to an institution, or death. 

To ensure a reasonably good response rate from the oldest old, the sample was stratified by 40% in the ages 67 to 79 years and 60% in the ages 80 and older. The results are weighted by gender and age, according to public demographic statistics (Statistics Norway).

### 2.2. Measures

Of interest is the frequency with which a respondent participates in various out-of-home activities. The set of activities included in the survey was meant to cover most of the daily activities of people past retirement age (67 years and older): grocery shopping, shopping for other goods, doing errands (bank, post office, pharmacy), health care (doctor, dentist, physiotherapist), recreational outdoor walking, doing (organized) exercise (indoors), visiting friends and family, attending meetings in organizations or clubs, and attending the cinema, theatre, concerts and/or restaurants and cafés.

The respondents were asked: How often do you do activity X?

- almost every day
- at least once a week
- at least once a month
- less than once a month
- never/not relevant

**Figure 1:**
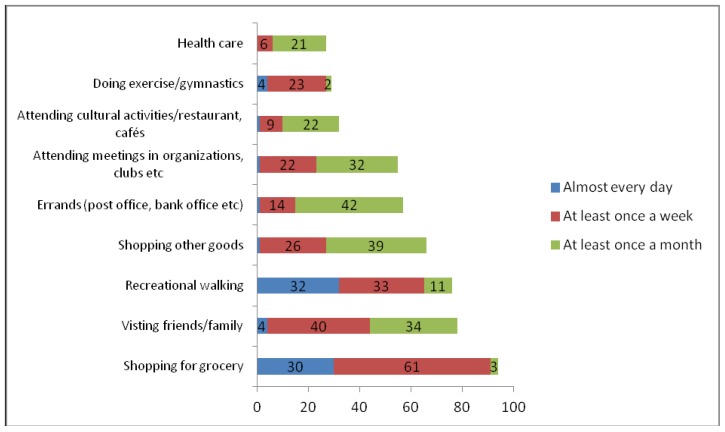
Trip frequency to activities in daily life (%). The number of valid answers varies from 4037 to 4341. Dependent variables are studied: overall mobility level (the sum of degree of participation in all the nine activities), degree of grocery shopping, degree of visits to family and friends, and degree of participation in cultural activities (cinema, theatre, concerts and/or restaurants and cafés).

The degree of participation in the investigated activities is shown in Figure 1. 

The selection of independent variables is guided by the theoretical framework outlined in Section 2. Table 1 gives an overview of the independent variables applied in the analysis and which dimensions they are meant to target. 

Health status was assessed through respondents’ own evaluations of their health by asking: “How do you consider your health in general?” The possible answers were “very good”, “good” “neither good nor poor”, “poor” and “very poor”. As previous research has shown that there is a strong association between self-reported health and objective health measures [37], we can assume that the subjective health reported in this study is linked to the respondent’s actual health. Ability to move was measured by the question: “Do you have health related problems with walking?” The respondents could choose: “large problems”, “some problems”, “no problems” or “not relevant”.

To assess whether or not a lack of social network could be associated with participation in a given activity, respondents were asked if lacking people to attend activities with was a reason for not attending more activities (“yes”/”no”). 

In this study, the degree of home orientation is applied as an indicator of preferences for indoor activities. To measure home orientation, the respondents were asked if they agreed that: “I prefer to stay at home, hence I do not have a great need for activities outside the home”. The alternatives were “largely agree”, “agree to a certain extent”, “do not agree”, and “don’t know”. Degree of home orientation is only weakly correlated with age (Parsons r = 0.261).

### 2.3. Statistical Analysis

Responses to degree of participation in different activities (nine items) were scored: “almost every day” = 5, “at least once a week” = 4, “at least once a month” = 3, “less than once a month” = 2, and “never/not relevant” = 1. 

In order to further analyse factors associated with overall level of out-of-home activity participation, the responses to the nine items assessing participation in an activity were arranged on a scale that indicates overall level of out-of-home activity participation: the higher the scale sum, the higher the degree of out-of-home activity participation. This scale was transformed into an index of the mean scale scores of each respondent; a score of 5 indicates participation in all nine activities almost every day, and 1 indicates that respondent never participates in any of the nine activities and/or that they are not relevant to this respondent. The mean score of this index is 2.88 (S.D. = 0.58), which indicates that, in general, each of the nine activities are frequented approximately at least once a month. The number of respondents included in this index is 4600. 

Linear regression analyses (method: enter) were applied to assess the factors associated with overall participation level and with the level of participation in three specific activities (grocery shopping, visiting friends and family, attending cultural activities/restaurants, etc.). Two models were explored to develop a deeper understanding of the associations between the independent variables and the overallg participation level among older people:

Model I: Individual resources and abilities for mobility and contextual conditions;

Model II: Individual resources and abilities for mobility, contextual conditions and degree of home orientation. 

To gain a deeper understanding of factors associated with, respectively, the degree of grocery shopping, the degree of visits to family and friends and the level of participation in cultural activities, one model was explored equivalent to Model II for the overall participation level. 

To avoid multicollinearity, the variable indicating having access to a car in the household was not included in the models, because it is highly correlated with holding a driving license (Pearson’s r = 0.69). It is assumed that holding a driving license is a slightly better measure of mobility options than access to a car in the household: 92.5% of those with a driving license have access to a car in the household and 90% of those who have access to a car in the household hold a driving license. 

Preliminary analyses of the independent variables were performed and these showed no violation of the assumptions regarding normality, linearity, multicollinearity, and homoscedasticity. 

## 3. Results

### 3.1. Overall Level of out-of-Home Activity Participation

Table 2 presents the descriptive analysis of older people’s mean scores on the overall out-of-home activity participation index across different levels of the independent variables. One-way ANOVA analyses were conducted to explore whether there are significant associations between the overall out-of-home activity participation level and various independent variables.

The results of these analyses show a significant association between overall participation level and all of the independent variables, except gender. See Table 2. 

As expected, there is a significant difference between the oldest old (80 years and older) and the younger old (67 to 79 years). Those living with a spouse/partner have a significantly higher level of out-of-home activity participation than those who do not. Level of participation tends to increase with income, educational level, holding a driving license, access to a car in the household, higher degree of centrality of residence, and quality of the public transport supply (in terms of both frequency and distance to the nearest stop). Out-of-home activity participation tends to decrease with poorer overall health and with greater physical problems with walking. Those who have agreed with the statement “I have too few people to attend activities with” have a significant lower level of out-of-home activity participation than those who did not agree. 

The level of out-of-home activity participation decreases significantly with a higher degree of home orientation, and analyses show that home orientation varies with background factors and individual ability for mobility. More men than women (37% to 32%), report that they agree to a large degree that: “I prefer to stay at home, and do not have a great need for activities outside the home”; a larger share of those age 80 or older report this than those who are younger (48% to 28%), a larger share of those who have poor health compared to those who have poor or neutral health report the same (42% to 34%), and those who report large problems with walking are more prone to agree to a large degree on this question than those who have no problems with walking (45% to 26%). A larger share of those who do hold a driver’s license and those who do not have access to a car in the household agree to “a large degree” that they are home oriented (respectively, 47% to 29%). All these differences are significant.

Because the independent variables in the bivariate analysis are likely to be correlated, multivariate analyses have been undertaken that allow the unique contribution of each independent variable to be identified when explaining the variations in the level of out-of-home activity participation. Even though the direction of causation cannot be interpreted from the following multivariate analyses, they give important insights into the strength of the association between an independent variable and the dependent variable, when other independent variables are controlled for. 

Two models were estimated: Model 1 estimates the associations between the independent variables and the overall level of out-of-home activity participation when home orientation is not included, and Model 2 includes home orientation. These two models are presented in Table 3.

In Model 1, the most important correlates with overall participation level are, in order of importance, age, holding a driving license, physical problems with walking, and level of education. It is worth noting that the effect of living without a spouse/partner has reversed compared to the bivariate model and is now positive. This indicates that when differences in confounding variables are kept constant, older people living without a spouse/partner tend to undertake more out-of-home activities. 

Although we found no significant gender difference in overall participation level in the bivariate analysis, the multivariate analysis suggests that being a woman has a significant and positive effect on theoverall participation level, but the effect is relatively small compared to that of other significant variables. Frequency of public transport service also has a positive effect on the level of out-of-home activity participation. Moreover, access to company during activities (“I have too few people to attend activities together with”) has a significant but minor effect.

Model 2 includes degree of home orientation, and all of the independent variables except gender have a significant effect on the overall participation level, which suggests that the gender effect found in Model 1 might be explained by a larger degree of home orientation among older men than older women. 

Moreover, the effects of many of the independent variables included in Model 1 are reduced for the same variables in Model 2. This reduction is especially large for age, living status, income, educational level, and frequency of public transport supply. This suggests that home orientation is most prevalent among the oldest old, those living with a spouse/partner, those with a lower income and educational level, and those with a poor public transport supply. The effects of holding a driving license, health condition, and physical problems with walking remain more or less constant from Model 1 to Model 2; this implies that there are not many interdependencies among these variables and degree of home orientation. 

Model 2 suggests that age, holding a driving license, and physical problems with walking are among the variables with the strongest associations with mobility level, but home orientation shows the highest effect. The strong association between home orientation and mobility level can also be read from the large rise in the adjusted R square from Model 1 (0.205) to Model 2 (0.286). The models estimated here are not causal models. The direction of causation cannot be interpreted from this.

### 3.2. Degree of Grocery Shopping, Visiting Family and Friends, and Cultural Activities

A standard multiple regression analysis was performed to assess the effect of various factors associated with respectively the degree of grocery shopping, visiting friends and family, and attending cultural activities. See Table 4. 

The results suggest that there are some major differences in the factors that influence the degree of participation in these three activities.

Being female has a relatively small positive impact on the tendency to visit family and friends and to attend cultural activities, but has no effect on the inclination to shop for groceries. 

The most important factors associated with the degree ofgroceryshopping are suggested to be age, health condition, and holding a driving license. Centrality of residence also has a significant effect on the level of this activity, which suggests that shorter distances and a greater supply of shops increases the propensity for grocery shopping. The frequency of public transport, too, has a significant effect on grocery shopping. 

The data show that the share of trips made by walking is greater for grocery shopping (27%) than for visiting family and friends (6%) and attending cultural activities (10%), which suggests that the distances are shorter to grocery stores than to family and friends and cultural activities.

Visiting family and friends is primarily associated with health condition, holding a driving license, and degree of home orientation. The effect of age is small, which suggests that the need for social contact and visits remains more or less constant throughout the ageing process. 

The major factors associated with attending cultural activities are age and degree of home orientation. Also, living without a spouse/partner increases the frequency of participation in cultural activities.

Holding a driving license has no effect on the frequency of cultural activities. This suggests that there is less need for a car to attend these activities than for others, which might be explained by better public transport supply into the city center, where these activities are often located. This is supported by the observation that the share of public transport use for trips related to cultural activities (19%) is significantly higher than for trips related to visiting family and friends (4%) and grocery shopping (10%). Moreover, the trips made by car (as a driver or a passenger) for cultural activities (56%) are significantly fewer than for trips to family and friends (78%) and grocery shopping (64%). The high share of car use on visits to family and friends suggests that the need for a car on these trips is greater than for grocery shopping and for cultural activities, which might be explained by family and friends being more geographically dispersed than the two former activities. 

Although both education and income levels have a positive impact on cultural activities, they do not have any effect on the frequency of grocery shopping or visiting family and friends. This probably relates more to the nature of these activities than to the mobility options/constraints on the travel to these activities: participation in cultural activities often requires a certain level of wealth (e.g., tickets to concerts and theatres, dining out at restaurants). One can also assume that increased wealth could improve the mobility options for participation in cultural activities, as it offers greater potential for the use of taxis or parking (often expensive parking lots in central areas) close to chosen activities. Moreover, participation in these kinds of activities might be an expression of a specific lifestyle and taste related to income and education [38], whereas grocery shopping and visiting family and friends are common to all. 

“I have too few people to attend activities together with” has a negative effect on visits to family and friends and on cultural activities, but no effect on the frequency of grocery shopping, which probably reflects that the former are more social activities than the latter. 

## 4. Discussion

This study of a random sample of older adults in Norway indicates that older people’s overall needs fulfilment, as measured by the level of out-of-home activity participation, is positively linked with living alone, good health, no physical problems with walking, and financial resources, and negatively linked with home orientation, not holding a driving license, lack of company on activities, living in a rural area, and poor quality of public transport supply.

One of the main objectives of this study was to explore whether needs fulfilment, as measured by out-of-home activity participation in old age, is a result of both preferences and opportunities for mobility. Preferences might be shaped by increasing constraints on mobility in old age. Nevertheless, when controlling for various individual and contextual constraints for mobility, the findings suggest that the degree of home orientation plays an important role for the overall level of out-of-home activity participation in old age. This finding indicates that older people are different in their preferences for out-of-home activities (independent of age), and that they will choose differently within the same opportunities for mobility, which is in line with Elster’s approach to action. Furthermore, the study also shows that degree of home orientation has an independent effect on the extent of social visits and cultural activities when controlling for other factors, which might indicate that people have different preferences for these kinds of activities. Degree of home orientation is, however, less important for the amount of grocery shopping, which might indicate that the degree of grocery shopping is more independent of preferences, as groceries are a common need to all. One could argue that applying only one indicator of preference (degree of home orientation) is too limited. The purpose of this study was not to capture preferences for different types of activities, e.g., preferences for active outdoor activities, cultural activities, social activities, etc. In such a case, several measures would have been necessary. However, the focus of this study has been on degree of out-of-home activities, and it is reasonable to believe that differences in degree of home orientation reflects differences in preferences for in-home activities rather than out-of-home activities. Nevertheless, it is likely that older people are just as different as the rest of the population in terms of preferences for out-of-home activities, and that this will affect where and how often they travel, and which out-of-home activities they participate in. Lifestyle is a useful concept to capture variations in peoples’ preferences and actions [38,39]. One future avenue of future research could be identifying specific lifestyles among older people, and their related mobility and needs fulfilment. Based on such research, policies can be developed that target the mobility challenges in the different lifestyles of older persons. 

Notwithstanding, the findings also suggest that needs fulfilment and out-of-home activity participation in old age is, to a large degree, constrained, including among those who have a low degree of home orientation. The findings in this study support the studies of Siren and Hakamies-Blomqvist [28] and Marottoli et al. [11]: holding a driving license is of major significance forout-of-home activity participation in old age. Even when controlling for home orientation, the relative importance of holding a driving license remains more or less the same. This suggests that policies should be developed and implemented to maintain older people’s ability to drive, such as refresher courses and other encouragements for continued driving. Alternatively, for older people who are unable to hold a driving license anymore, and who are not able to use the public transport system, policies should be developed to secure their mobility to desired activities through, e.g., a special car transport service. Moreover, our study suggests that income affects out-of-home activity participation: keeping a car in old age might be difficult, as income decreases after retirement or the loss of a spouse. This calls for social policies that help older people keep a car in the household. In addition, income might not only prevent older people to travel to an activity, but also from being able to actually participate in an activity, as this may be too costly (e.g., cultural activities). The effect of income on overall participation level suggests that there are social inequalities among older people in terms out-of-home activity participation.

This study found that holding a driving license is not associated with level of participation in cultural activities. This might be explained by a reluctance to drive into the city center, where cultural activities are often located. Focus group interviews from Norway have shown that older people are often reluctant to travel by car into the city center because parking is expensive, or they worry about the traffic [40]. However, as suggested in the result section, better financial resources might improve the mobility options for cultural activities, by providing for expensive parking or for taxis. Moreover, there is usually a better public transport supply into city-center activities than to those that require crisscross travels. As observed in this study, the share of public transport use is greater for cultural activities than for visiting family and friends (who are often dispersed across the city). These results indicate that there is a potential in urban planning to increase public transport use in old age by clustering activities around key public transport connection points. Both public and private actors that offer activities for older people should carefully consider the location of these activities relative to the public transport supply. However, the public transport sector itself has to considerably improve the quality of its services. Previous research has shown that older people experience a range of barriers to using public transport [41,42,43,44,45] such as long distances to stops, problems entering and leaving a vehicle, fear of crime during evening travel, and lack of seating. Poor provision of services at night and on the weekends has been found in other qualitative studies to constrain mobility in old age [8,40]. 

We hypothesized that holding a driving license would be of minor importance for the frequency of grocery shopping, because grocery stores are often located in neighbourhoods, within walking distance of homes. Although our study shows that centrality of residence, where the supply of grocery stores is greater and the distances shorter, is associated with the frequency of this activity, holding a driving license is identified as one of the most important factors for explaining variations in the level of these trips. Other findings from qualitative studies indicate that older people use the car for grocery shopping to avoid carrying heavy loads [40,42] and that older people who can no longer drive combat this problem by making more frequent trips. The link between car use and grocery shopping in central areas where grocery stores are often located in the neighbourhood should be examined more closely: What is perceived as a tolerable walking distance? What are the main barriers to walking? Is there a potential to promote more walking? More walking in daily life could lead to greater health benefits and keep older people independent for longer. 

The study suggests that age has an independent effect on needs fulfilment as measured by out-of-home activity participation. It is difficult to interpret this age-effect, especially when constraining factors are controlled for. The finding might suggest that there are other potential constraining factors that have not been explored in this study, such as a reduction in the social network of family and friends that can act as chauffeurs as people age, or that the number of trips by special car transport services is insufficient. Having the possibility to use the special car transport service is especially important, as health conditions and other physical problems often increase with age and make it difficult to walk, to use public transport and to drive a car. 

In line with previous research, the findings in this study clearly demonstrate that there are transport policy challenges for improving mobility in old age. However, this study expands on previous research by suggesting that the transport policy challenges differ between various types of activities, the location of an activity, its accessibility by public transport, and its nature. Finally, and notwithstanding, the findings suggest that constraints on mobility and out-of-home activity participation in later life cannot be tackled only, and not even primarily, through transport planning: in order to improve mobility - and needs fulfilment - in later life, there is a need for a cross-sectoral approach that includes transport planners in close conjunction with health and social care professionals, and policy-makers, urban planners, and private/public operators of leisure activities.

## 5. Conclusions

The main conclusion from this study is that the extent to which out-of-home activities fulfill needs vary between individuals, depending on their preferences as well as the interplay between individual abilities and resources and contextual conditions. 

## Figures and Tables

**Table 1:** Overview of independent variables and the dimensions they target.

Independent Variables/Dimensions	Background Factors	Preferences	Individual Resources and Abilities for Mobility	Contextual Conditions for Mobility
Age	X			
Gender	X			
Living status		X	X	
Income	X		X	
Education	X			
Physical problems with walking			X	
Self-evaluation of health condition			X	
Have too few people to attend activities together with			X	
Access to a car in the household			X	
Holding a driving license			X	
Centrality of residence				X
Distance to the closest public transport stop				X
Frequency of the closest public transport service				X
Home orientation		X		

**Table 2:** Mean level of out-of-home activity participation (overall level of out-of-home activity participation index) by individual and contextual mobility options and by degree of home orientation.

Independent Variable	Value	Mean	*N*	Std. Deviation
Gender ^n.s.^	Male	2.8991	1955	0.5229
	Female	2.8689	2617	0.6151
Age ***	Age 67–79	2.9559	2999	0.5055
	Age 80 or older	2.7382	1593	0.6711
Living status ***	Living with a spouse/partner	2.9245	2271	0.5057
	Not living with a spouse/partner	2.8391	2058	0.6216
Education level ***	Primary school	2.7604	1756	0.6089
	Secondary school: vocational training	2.8579	872	0.4991
	Secondary school: theoretical training	2.9461	320	0.5832
	University/college	3.0819	969	0.4606
Household income ***	Less than 200,000 NOK	2.7110	1063	0.6581
	200,000–399,000 NOK	2.8878	1841	0.5303
	400,000–599,000 NOK	2.9856	919	0.4630
	600,000 NOK or more	3.1166	416	0.4697
Car in the household ***	Yes	2.9544	3037	0.4878
	No	2.7075	1109	0.6755
Driving license ***	Yes	2.9743	3003	0.4750
	No	2.6757	1249	0.6856
Centrality of residence ***	City/town centre	2.9375	587	0.6071
	Urban area, middle part	2.9093	891	0.5672
	Urban area, periphery	2.9198	1769	0.5557
	Rural area	2.7848	1123	0.5569
Public transport: Frequency ***	Several departures per hour	2.9532	1555	0.5419
	Once per hour	2.9538	866	0.5142
	Less often	2.8056	1171	0.5482
Public transport: Distance to stop ***	Less than 200 m	2.8803	1241	0.5739
	200–499 m	2.9361	1592	0.5403
	500–999 m	2.8679	734	0.5421
	1000 m or more	2.8126	630	0.5442
Physical problems with walking ***	Big problems	2.5097	334	0.6661
	Some problems	2.8667	872	0.5139
	No problems	3.0086	2181	0.4346
Health condition ***	Good	2.9895	2864	0.4796
	Neither good nor poor	2.7753	1135	0.5838
	Poor	2.4654	411	0.7740
I have too few people to attend activities together with ***	-	2.8924	4018	0.5806
	+	2.8010	583	0.5518
Home orientation ***	Agree to a large extent	2.6340	1353	0.5817
	Agree to some extent	2.8982	1470	0.4771
	Disagree	3.1357	1119	0.4594

*** *p* < 0.001, n.s. = *p* > 0.05 (Anova, F-test).

**Table 3:** Results from two standard regression models ^1^ of the overall level of out-of-home activity participation among older people.

Independent Variables	Model 1 ^2^		Model 2 ^3^	
	(*N* = 2092)		(*N* = 2092)	
	Beta	Sig.	Beta	Sig.
(Constant)		0.000		0.000
Gender (female)	**0.061**	0.007	0.024	0.268
Age in years	**−0.151**	0.000	**−0.095**	0.000
Living status (living without a spouse/partner)	**0.103**	0.000	**0.076**	0.002
Income (low–high)	**0.089**	0.001	**0.056**	0.034
Education level (low–high)	**0.129**	0.000	**0.076**	0.001
Driving license (“No”)	**−0.138**	0.000	**−0.121**	0.000
Centrality of residence (urban–rural)	−0.041	0.060	**−0.051**	0.013
Public transport: Distance to stop (short–long)	−0.024	0.240	**−0.044**	0.026
Public transport: Frequency (often–seldom)	**−0.097**	0.000	**−0.057**	0.007
Physical problems with walking (“None”) ^4^	**0.130**	0.000	**0.114**	0.000
Health condition (“Good or neutral”) ^4^	**0.097**	0.000	**0.099**	0.000
Have too few people to attend activities together with (“Yes”)	**−0.045**	0.023	**−0.049**	0.009
Home orientation (Disagree–agree)			**−0.311**	0.000

^1^ For ease of reading, betas which are significant (*p* < 0.050) are marked in bold. ^2^ Adjusted R square = 0.205, F(12/2079) = 45.81, *p* < 0.001. ^3^ Adjusted R square = 0.286, F(13/2078) = 65.5, *p* < 0.001. ^4^ Coded as dichotomous variables.

**Table 4:** Results from regression models ^1^ for the degree of grocery shopping, visiting family and friends, and attending cultural activities among older people.

Independent Variables	Grocery Shopping ^2^		Visiting Family and Friends ^3^		Attending Cultural Activities ^4^	
	(*N* = 2049)		(*N* = 2026)		(*N* = 2055)	
	Beta	Sig.	Beta	Sig.	Beta	Sig.
(Constant)		0.000		0.000		0.000
Gender (female)	−0.034	0.161	**0.055**	0.023	**0.072**	0.002
Age in years	**−0.109**	0.000	**−0.058**	0.022	**−0.133**	0.000
Living status (not living with partner or spouse)	0.010	0.721	0.049	0.074	**0.081**	0.002
Income (low–high)	−0.011	0.706	0.021	0.489	**0.064**	0.023
Education (low–high)	0.023	0.363	0.004	0.885	**0.097**	0.000
Driving license (“No”)	**−0.106**	0.000	**−0.090**	0.001	−0.032	0.184
Centrality of residence (urban–rural)	**−0.093**	0.000	−0.009	0.704	**−0.051**	0.019
Public transport: Distance to stop (short–long)	−0.018	0.417	**−0.054**	0.014	−0.011	0.592
Public transport: Frequency (often–seldom)	**−0.085**	0.000	**0.071**	0.003	**−0.065**	0.004
Physical problems with walking (“None”)	**0.072**	0.003	**0.049**	0.050	**0.050**	0.030
Health condition (“Good or neutral”)	**0.152**	0.000	**0.122**	0.000	**0.086**	0.000
Have too few people to attend activities with (“Agree”)	0.024	0.257	**−0.086**	0.000	**−0.047**	0.019
Home orientation (disagree–agree)	**−0.070**	0.002	**−0.211**	0.000	**−0.251**	0.000

^1^ For ease of reading, betas which are significant (p < 0.050) are marked in bold. ^2^ Adjusted R square = 0.127 (F = 13/2035) = 23.86, *p* = 0.001. ^3^ Adjusted R square = 0.114 (F = 13/2012) = 21.12, *p* = 0.001. ^4^ Adjusted R square = 0.208 (F = 13/2041) = 25.07, *p* = 0.001.
